# *E*- and *Z*-Isomers of New Phytoecdysteroid Conjugates from French Polynesian *Microsorum membranifolium* (Polypodiaceae) Fronds 

**DOI:** 10.3390/molecules171011598

**Published:** 2012-09-28

**Authors:** Raimana Ho, Jean-Pierre Girault, Phila Raharivelomanana, René Lafont

**Affiliations:** 1 Laboratoire Biodiversité Terrestre et Marine, Université de la Polynésie Française, B.P. 6570 Faa’a, 98702 FAA’A, Tahiti, French Polynesia; 2 Laboratoire de Chimie et Biochimie Pharmacologiques et Toxicologiques, CNRS UMR 8601, Université Paris Descartes, 45 rue des Saints Pères, 75270 Paris, Cedex 06, France; 3 Laboratoire BIOSIPE, ER3, Paris 6 – Pierre et Marie Curie, Case 29, 7 Quai Saint Bernard, 75252 Paris, Cedex 5, France

**Keywords:** phytoecdysteroids, fronds, *Microsorum membranifolium* (R. Br.) Ching, Polypodiaceae, French Polynesia

## Abstract

Phytochemical investigation of the fronds of *Microsorum membranifolium* resulted in the isolation of a new phytoecdysteroid, *E*-2-deoxy-20-hydroxyecdysone 3-[4-(1-β-D-glucopyranosyl)]-caffeate (**1**), together with two known phytoecdysteroids, *E*-2-deoxy-20-hydroxyecdysone 3-[4-(1-β-D-glucopyranosyl)]-ferulate (**2**), *E*-2-deoxyecdysone 3-[4-(1-β-D-glucopyranosyl)]-ferulate (**3**). Their respective *Z*-isomers **4**–**6** were also observed and identified for the first time. The new structures were elucidated on the basis of extensive spectroscopic data analysis (1D, 2D-NMR and HR-MS techniques).

## 1. Introduction

**Figure 1:**
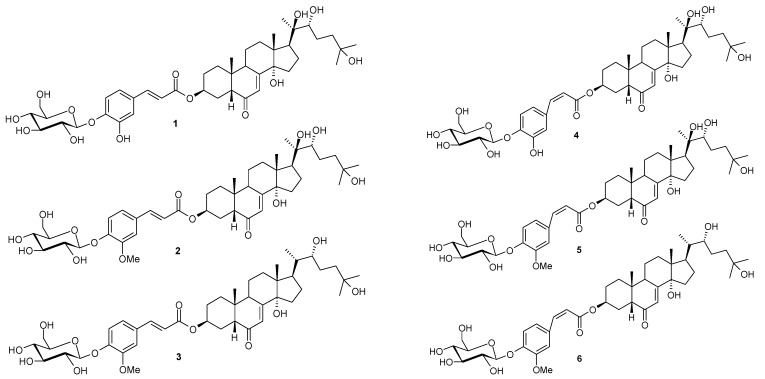
Chemical structures of phytoecdysteroids **1**–**6** from *M. membranifolium*.

*Microsorum membranifolium*, which belongs to the Polypodiaceae family, is one of the most frequently used fern species in Polynesian traditional medicine. The fronds and/or the rhizomes of *M. membranifolium*, named “Metuapua’a” in French Polynesia, are usually prescribed in popular remedies to treat stomach ache, gonorrhoea, pneumonia, leucorrhoea, sterility, dislocations and fractures [1,2,3]. This plant contains phytoecdysteroids as main bioactive components, including ecdysone, 20-hydroxyecdysone, 2-deoxy-20-hydroxyecdysone and 2-deoxyecdysone [4]. Previous phytochemical investigation of the fronds of the medicinal fern *M. membranifolium* revealed a new class of phytoecdysteroid conjugates [5]. As a part of a continuing project to study this new class of phytoecdysteroid conjugates, we investigated the BuOH fraction of the fronds of this medicinal fern. A new phytoecdysteroid, *E*-2-deoxy-20-hydroxyecdysone 3-[4-(1-β-D-glucopyranosyl)]-caffeate (**1**), together with two known phytoecdysteroids, *E*-2-deoxy-20-hydroxyecdysone 3-[4-(1-β-D-glucopyranosyl)]-ferulate (**2**), *E*-2-deoxyecdysone 3-[4-(1-β-D-glucopyranosyl)]-ferulate (**3**), and their respective *Z*-isomers **4**–**6** were also observed and identified for the first time. In this paper, we present the isolation and structural determination of these phytoecdysteroids (**1**–**6**, Figure 1) from *Microsorum membranifolium* (R. Br.) Ching.

## 2. Results and Discussion

The chemical investigation of the butanolic extract of the fronds of *Microsorum membranifolium* led *E*-2-deoxy-20-hydroxyecdysone 3-[4-(1-β-D-gluco-pyranosyl)]-caffeate (**1**), along with two known phytoecdysteroids **2** and **3** and their respective *cis* isomers **4**, **5** and **6**.

Compound **1** was obtained as a white amorphous powder. The mass spectrum was consistent with a M.W. of 788 amu. The UV spectrum shows a typical ecdysteroid spectrum, with a maximum at 240 nm (in EtOH), but in addition to the major absorbance at 240 nm, peaks were observed at 290 and 313 nm, which are indicative of the presence of an aromatic conjugating moiety. Initial examination of the ^1^H-NMR spectrum of this compound shows that it presents an ecdysteroid aglycone conjugated with a glycoside sugar. The presence of a sugar is straightforward, since one observes additional peaks in the region of hydrogen bound to oxygenated carbons (3.2–4.95 ppm) and the corresponding carbon signals (60–105 ppm) in the ^13^C-NMR spectrum. On the other hand, we could note the presence of a slightly different aromatic moiety, but with a relatively similar spin system with respect to ferulate compounds **2** and **3** [5]. However, this aromatic group presents the loss of the methyl signal of the methoxy group present in ferulate [4,5]. This could be consistent with a caffeate structure for this aromatic moiety. The NMR spectroscopic evidence below confirmed this assignment. Inspection of the ^1^H-NMR of the ecdysteroid core of this molecule shows five singlet methyl signals for compound **1**. This is typical of 20-hydroxy-derivatives, which was also confirmed after assignment of ^1^H or ^13^C signals by means of 1D and 2D experiments. Moreover this compound does not show significant changes in its ^1^H or ^13^C chemical shifts for the signals of the side-chain or of rings B, C and D of the ecdysteroid core. However, as for the A-ring of 2-deoxy-20- hydroxy-ecdysone 3-[4-(1-β-D-glucopyranosyl)]-ferulate (2), we observe the typical features of 2-deoxy compounds (lack of H-2 in the >CHOH zone, broadening of Η_eq_-3 and of 3-esterified derivatives (Η_eq_-3: δ = 5.11 ppm, broad singlet, w_1/2_ = 13 Hz) [6,7]. Finally, examination of the ^1^H and ^13^C spectral data of the conjugated moieties led to the identification of the aromatic moiety as a caffeate and for the sugar moiety as a β-D glucoside as follows [8]: (**i**) the sugar presents one oxymethylene and five oxymethine groups in agreement with a hexose sugar; this hexose presents a ^1^H anomeric NMR signal H-1' at δ = 4.86 ppm in agreement with a 1'-glycosidic link to the rest of the molecule. On the other hand, the large ^3^*J* coupling (doublet, 7.3 Hz) is in accordance with a H_axial_-H_axial_ coupling constant and consequently with an axial position of both H-1' and H-2' protons. ^1^H selective homonuclear decoupling experiments for H-1', H-6' and H-6" and examination of 2D TOCSY cross-peaks show clearly that H-3', H-4' and H-5' are in axial positions owing to the large ^3^*J* coupling constants of H-2'-H-3', H-3'-H-4' and H-4'-H-5'. 2D ROESY experiments present ROE correlations in agreement with this conclusion, so we conclude that sugar moiety is a β-D glucoside; (**ii**) the linkage of this β-D glucoside was deduced from 2D HMBC experiments as one observes a correlation from H-1' to a quaternary carbon of the aromatic part of the molecule. This shows that the glucoside is linked with the aromatic moiety, which is itself linked to the ecdysteroid core moiety by an ester bond. This is confirmed from a ROE correlation observed for H-5 (δ = 2.38 ppm) with H-8" (δ = 6.40 ppm). No correlation could be observed from 2D HMBC experiment for the broad H-3, as this signal has unfavorable relaxation properties; (**iii**) the aromatic moiety presents ^1^H and ^13^C spectral data of an ester group linked to a double bond bearing two ethylenic protons in *trans*-configuration (unambiguously established from the value of their large [16.1 Hz] 3J coupling constant).

**Figure 2:**
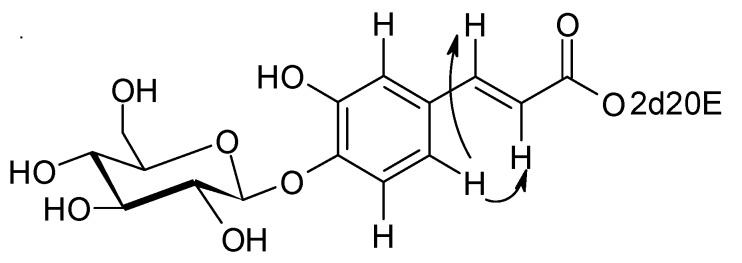
Selected ROE correlations of compound **1**.

Again, from 2D HMBC experiments, this double bond could be linked to a phenyl ring bearing three protons, a hydroxy group and the glucosidic link with the sugar (see above). The aromatic proton at δ = 7.14 ppm (d, *J* = 1.8) presents ROE correlations with the two ethylenic protons H-7" and H-8" (Figure 2). All these elements together with MS data are in agreement with a caffeate structure for this aromatic group. In conclusion, compound **1** corresponds to *E*-2-deoxy-20-hydroxyecdysone 3-[4-(1-β-D-glucopyranosyl)]-caffeate.

The two other known compounds were identified as *E*-2-deoxy-20-hydroxyecdysone 3-[4-(1-β-D-glucopyranosyl)]-ferulate (**2**) [5] and *E*-2-deoxyecdysone 3-[4-(1-β-D-glucopyranosyl)]-ferulate (**3**) [5], by interpretation of their spectroscopic data.

In NMR spectra of compounds **1**, **2** and **3**, we could note the presence of signals of other compounds (**4**, **5** and **6**) very similar to compounds **1**, **2** and **3** (with ratio of ca 20% after initial dissolution in CD_3_OD and 55% after 1 month), but which only present main differences in the signals of the ferulate moiety for **2** and **3** and of the caffeate moiety for **1**. These added signals correspond to the presence of ethylenic double bound *cis* (*Z*)-isomer compounds of ferulate and cafeate in mixtures with compounds **1**, **2** and **3**, which have a *trans* (*E*)-configuration of the ethylenic double bond. This *cis*-configuration led to a significant variation of chemical shift of the ethylenic proton signals H-7" and H-8". The value of 12.8 Hz for ethylenic coupling constants ^3^*J* H-7"-H-8" is in agreement with this *cis*-configuration. We could note that for their respective *trans*-isomers, a value of 16.1 Hz is observed for their ^3^*J* H-7"-H-8" coupling constants in agreement with their *trans* (*E*)-configuration of their double bond. Moreover the *cis* configuration of these compounds is confirmed thanks to the large NOE effect observed between ^1^H signal of H-7" and H-8". Ferulate and caffeate compounds belong to the family of cinnamic compounds derivatives for which thermodynamic equilibrium of *cis*-isomer with its *trans*-isomer is a well-known phenomenon [9,10]. Although the *trans*-*cis*-equilibrium is common, this is the first time that the *cis*-isomers **4**–**6** in this new class of ecdysteroid conjugates (glycosyl-ferulates and -caffeates) have been observed and identified.

Three analytical high performance liquid chromatography (HPLC) methods have been developed for the separation of isomers of these phytoecdysteroids. We observed that the elution order of the *Z*-isomers was reversed in comparison with the *E*-isomers between the two normal-phase HPLC systems (System B and C). We also noted that the isomers eluted in the same order with the systems A (RP) and B (NP). With the system C, compound **2** eluted surprisingly much later than compound **1**. This result was unexpected as compound **1** should be more polar than **2** because of its free hydroxy instead of a methoxy group. Chromatographic data are reported in Table 1.

**Table 1:** HPLC data for the different phytoecdysteroids.

Phytoecdysteroid	Retention time (min)		
	System A	System B	System C
**1**	25.7	15.9	15.6
**2**	26.7	12.3	17.6
**3**	34.0	9.2	14.2
**4**	32.0	25.2	13.5
**5**	33.5	19.2	16.0
**6**	41.0	12.7	12.7
20-hydroxyecdysone	7.5	16.0	9.4
2-deoxy-20-hydroxyecdysone	15.4	8.3	6.0
2-deoxyecdysone	24.1	6.1	5.0

## 3. Experimental

### 3.1. General

UV spectra were recorded in EtOH with a Varian DMS 100 spectrometer. The NMR spectra were obtained on a Bruker Avance 500 (Wissembourg, France) at 300 K. The samples were lyophilized in D_2_O and dissolved in CD_3_OD. ^1^H signal of residual CHD_2_OD (3.31 ppm) and of ^13^C signal of ^13^CD_3_OD (49.0 ppm) were used as internal reference respectively for proton and carbon shifts (δ ± 0.2 ppm). Chemical shifts are expressed in ppm. 1D ^1^H and ^13^C spectra and 2D COSY, TOCSY, NOESY, ROESY, PFG-HSQC and PFG-HMBC NMR spectra further allowed the ^1^H and ^13^C assignments [6,11]. ESIMS were recorded on a Jeol JMS-700 spectrometer (Croissy sur Seine, France) either in desorption/chemical ionization (CI/D) mode or fast-atom bombardment (FAB) mode. HRESIMS data were obtained on a LTQ-Orbitrap-XL mass spectrometer. HPLC-UV-DAD analysis was carried out on a HP 1100 system equipped with a photodiode array detector (Agilent Technology, Palo Alto, CA, USA) with an Interchrom C_18_ column (250 × 4.6 mm). Preparative HPLC separation (HP 1100) was carried out on a Chromanorm C_18_ S5 ODS II (250 × 20 mm) and a Zorbax-SIL (250 × 4.6 mm) with a photodiode array detector. Polyamide DC 6 (50–160 mm, Fluka) was used for column chromatography. Thin-layer chromatography was performed on silica gel 60 F_254_ A1 sheets (Merck, Darmstadt, Germany) and spots were visualized under UV (254 nm).

### 3.2. Plant Material

Fronds of *Microsorum membranifolium* (R. Br.) Ching were collected in Tahiti (French Polynesia) in the district of Papenoo in May 2007 and were identified by Dr. Jacques Florence. A voucher specimen has been deposited at the Herbarium of the “Museum of Tahiti and its Islands” in Punaauia (PAP, Tahiti Island).

### 3.3. Extraction and Isolation

The dried, milled fronds of *M. membranifolium* (1.15 kg) were exhaustively extracted with ethanol (4 L) with continuous stirring over 2 days at room temperature. The extract was filtered and the filtrate evaporated under reduced pressure to yield a syrup-like residue (33.81 g). The syrup was partitioned between CHCl_3_, H_2_O and then *n*-BuOH to give 4.14 g, 20.93 g and 8.83 g portions, respectively. The BuOH fraction was chromatographed using a column of polyamide and elution with 25% EtOH–H_2_O yielded 10 fractions. Similar fractions were combined after TLC and HPLC examination to provide four fractions, A (1.28 g), B (0.6 g), C (2.25 g) and D (4.62 g). Fraction B was submitted to a preparative chromatography using C_18_ column and a stepwise mobile phase gradient of ACN/water to afford twelve sub-fractions B1–B12. Compounds **1** (1.5 mg, t_R_ = 23.9 min) and **4** (1.2 mg, t_R_ = 22.4 min) were obtained by preparative normal-phase HPLC (Zorbax-SIL, 250 × 4.6 i.d.) of the sub-fraction B4 (2.7 mg; eluted with cyclohexane/isopropanol/water 100:50:3). Sub-fraction B6 (13.5 mg) eluted with dichloromethane/isopropanol/water (125:40:3) was separated on Zorbax-SIL, to afford **2** (4 mg, t_R_ = 10.8 min) and **5** (3 mg, t_R_ = 12.3 min). In addition, sub-fraction B10 (8.5 mg) was purified by normal preparative HPLC with dichloromethane/isopropanol/water (125:40:3) to give **3** (3 mg, t_R_ = 8.8 min) and **6** (2 mg, t_R_: 9.4 min).

### 3.4. Analytical HPLC Methods

HPLC equipment from Thermo was used for checking the separation of the isomers. Analytical reversed-phase (RP) HPLC was performed on an ACE C_18_ column (150 mm, 4.6 mm i.d., particle size 5 μm, from A.I.T.), eluted at a flow-rate of 1 mL/min with a linear gradient (15% to 35% acetonitrile-isopropanol [5:2 v/v] in water containing 0.1% trifluoroacetic acid in 40 min, then 35% to 100% in 30 min) (System A). Analytical normal-phase (NP) HPLC used an ACE 5 SIL column (150 mm, 4.6 mm i.d., particle size 5 μm, from A.I.T.), eluted at a flow-rate of 1 mL/min with dichloromethane-isopropanol-water (125:30:2, v/v/v) (System B) or with cyclohexane-isopropanol-water (100/40/3, v/v/v) (System C).

*E-2-Deoxy-20-hydroxyecdysone 3-[4-(1-β-D-glucopyranosyl)]-caffeate* (**1**): white amorphous powder; UV (EtOH) λ_max_ (log ε) nm: 242, 290, 313; HRESIMS (positive ion mode) *m/z*: 811.38726 [M+Na]^+^, (calc. for C_42_H_60_O_14_Na, 811.38753); ^1^H-NMR and ^13^C-NMR data are shown in Table 2 and Table 3.

*E-2-Deoxy-20-hydroxyecdysone 3-[4-(1-β-D-glucopyranosyl)]-ferulate* (**2**): white amorphous powder; UV (EtOH) λ_max_ (log ε) nm: 240, 294, 317; ESIMS (+) *m/z* 825 [M+Na]^+^, 841 [M+K]^+^; HRESIMS (positive ion mode) *m/z*: 825.40282 [M+Na]^+^, (calc. for C_43_H_62_O_14_Na, 825.40318); ^1^H-NMR and ^13^C-NMR data are shown in Table 2 and Table 3.

*E-2-Deoxyecdysone 3-[4-(1-β-D-glucopyranosyl)]-ferulate* (**3**): white amorphous powder; UV (EtOH) λ_max_ (log ε) nm: 240, 294, 317; ESIMS (+) *m/z* 825 [M+K]^+^, 809 [M+Na]^+^, 787 [M+H]^+^; ^1^H-NMR and ^13^C-NMR data are shown in Table 2 and Table 3.

**Table 2:** ^1^H-NMR data for compounds **1**–**6** in CD_3_OD (*δ* in ppm, *J* in Hz).

	δ_H_ (1)	δ_H_ (2)	δ_H_ (3)	δ_H_ (4)	δ_H_ (5)	δ_H_ (6)
Hax−1		1.46	1.46			
Heq-1		1.76	1.76			
Hax-2		1.87	1.87		1.77	
Heq-2		1.94	1.94			
Heq-3	5.16 (s, br, w_1/2_ = 13)	5.18 (s, br, w_1/2_ = 13)	5.16 (s, br, w_1/2_ = 14)	5.12 (s, br, w_1/2_ = 13)	5.11 (s, br, w_1/2_ = 13)	5.11 (s, br, w_1/2_ = 13)
Hax-4	1.77	1.77	1.77		1.77	
Heq-4	1.87	1.87	1.87		1.87	
H-5	2.38 (d, d, 12.3, 4.0)	2.40 (d, d, 12.3, 4.0)	2.41 (d, d, 12.6, 4.2)	2.15	2.2	2.2
H-7	5.84 (d, 2.1)	5.84 (d, 2.4)	5.85 (d, 2.0)	5.80 (d, 2.1)	5.81 (d, 2.1)	5.81 (d, 2.1)
Hax-9	3.28	3.27	3.27 (m, w_1/2_ = 26)	3.21	3.23	3.21
Hax-11	1.68	1.68	1.67		1.63	1.67
Heq-11	1.76	1.76	1.78		1.71	1.78
Hax-12	2.15	2.15 (t, d, 13.0, 4.8)	2.13 (t, d, 13.0, 4.8)	2.13	2.13	
Heq-12	1.86	1.88	1.78		1.87	
Hax-15	2.00	2.00	1.98		2.00	1.98
Heq-15	1.63	1.63	1.62		1.63	1.62
Hax-16	2.00	2.00	1.98	2.00	2.00	1.98
Heq-16	1.76	1.76	1.51	1.76	1.76	1.51
H-17	2.42 (t, 9.9)	2.42 (t, 9.9)	2.06	2.42	2.42	2.06
Me-18	0.908 (s)	0.907 (s)	0.745 (s)	0.880 (s)	0.890 (s)	0.735 (s)
Me-19	1.010 (s)	1.010 (s)	1.010 (s)	0.890 (s)	0.910 (s)	0.910 (s)
H-20			1.77			1.77
Me-21	1.203 (s)	1.203 (s)	0.957 (d, 6.6)	1.203 (s)	1.203 (s)	0.950 (d, 6.6)
H-22	3.36	3.35	3.61 (d, br, 10.1)	3.36	3.35	3.61
Ha-23	1.31	1.31	1.33	1.31	1.31	1.33
Hb-23	1.69	1.69	1.55	1.69	1.69	1.55
Ha-24	1.81	1.81	1.79	1.81	1.81	1.79
Hb-24	1.44	1.46	1.42	1.44	1.46	1.42
Me-26	1.197 (s)	1.197 (s)	1.200 (s)	1.197 (s)	1.197 (s)	1.195 (s)
Me-27	1.211 (s)	1.211 (s)	1.210 (s)	1.211 (s)	1.211 (s)	1.205 (s)
H-1'	4.86 (d, 7.3)	4.98 (d, 7.3)	4.98 (d, 7.3)	4.82 (d, 7.3)	4.96 (d, 7.3)	4.96 (d, 7.3)
H-2'	3.53	3.53 (d, d, 9.0, 7.5)	3.53 (d, d, 9.0, 7.5)		3.48	3.53
H-3'	3.48	3.48 (t, 9.0)	3.48 (t, 9.0)			3.48
H-4'	3.42	3.43 (d, d, 9.5, 8.6)	3.43 (d, d, 9.5, 8.6)			3.43
H-5'	3.46	3.45 (d, d, d, 9.5, 5.2, 2.2)	3.45 (d, d, d, 9.5, 5.2, 2.2)	3.46		3.44
H-6'	3.72 (d, d, 12.5, 5.3)	3.70 (d, d, 12.0, 5.0)	3.70 (d, d, 12.0, 5.0)	3.72 (d, d, 12.5, 5.3)	3.70 (d, d, 12.0, 5.0)	3.70 (d, d, 12.0, 5.0)
H-6'	3.91 (d, d, 12.2, 1.8)	3.89 (d, d, 12.2, 1.8)	3.89 (d, d, 12.2, 1.8)	3.91 (d, d, 12.5, 2.0)	3.89 (d, d, 12.2, 1.8)	3.89 (d, d, 12.2, 1.8)
H-2"	7.14 (d, 1.8)	7.31 (s, w_1/2_ = 3)	7.31 (s, w_1/2_ = 3)	7.15 (s, w_1/2_ = 3)	7.56 (s, w_1/2_ = 3)	7.56 (s, w_1/2_ = 3)
H-5"	7.21 (d, 8.6)	7.19	7.19	7.19 (d,8.6)	7.17 (d,8.6)	7.17 (d, 8.6)
H-6"	7.08 (d, d, 8.3, 1.8)	7.19	7.19	7.00 (d, d, 8.3, 1.8)	7.13 (d, d, 8.6, 1.8)	7.13 (d, d, 8.6, 1.8)
H-7"	7.60 (d, 16.1)	7.66 (d, 16.0)	7.66 (d, 15.8)	6.97 (d, 12.8)	6.97 (d, 12.8)	6.97 (d, 12.8)
H-8"	6.40 (d, 16.1)	6.50 (d, 16.0)	6.50 (d, 15.8)	5.90 (d, 12.8)	5.93 (d, 12.8)	5.93 (d, 12.8)
H-10"		3.92 (s)	3.92 (s)		3.87 (s)	3.87 (s)

Multiplicity of signals: s – singlet; d – doublet; t – triplet; m – multiplet; br – broad signal. w_1/2_: width at half-height in Hertz. ax: axial. eq: equatorial.

**Table 3:** ^13^C-NMR data for compounds **1**–**6** in CD_3_OD (*δ* in ppm, *J* in Hz).

	Multiplicity	δ_C_ (1)	δ_C_ (2)	δ_C_ (3)	δ_C_ (4)	δ_C_ (5)	δ_C_ (6)
C-1	CH_2_	30.1	30.1	30.1	30.1		30.4
C-2	CH_2_		26.3	26.3			26.3
C-3	CH	69.5	69.4	69.4	69.5	69.4	69.4
C-4	CH_2_		26.3	30.3			
C-5	CH	53.1	53.0	53.1	52.8	52.9	53.0
C-6	C	*	206.1	205.6	*	*	*
C-7	CH	121.8	121.7	121.5	121.8	121.7	121.5
C-8	C	*	*	*	*	*	*
C-9	CH	37.8	37.3	37.4	37.8		37.5
C-10	C		37.3	37.4			
C11	CH_2_		21.4	21.5			21.5
C12	CH_2_	32.4	32.5	32.0	32.4	32.5	32.1
C-13	C	49.0	49.0	48.2	49.0	49.0	47.4
C-14	C	85.6	85.4	85.4	85.6	85.6	85.4
C-15	CH_2_	31.6	31.7	31.6	31.6	31.7	31.6
C-16	CH_2_	21.4	21.4	26.9	21.4	21.4	26.9
C-17	CH	50.5	50.5	48.6	50.5	50.5	48.6
C-18	CH_3_	17.9	17.9	15.9	17.9	17.9	15.9
C-19	CH_3_	24.2	24.2	24.2	24.2	24.2	24.2
C-20	C	77.7	77.7	43.3 CH		77.7	43.3 CH
C-21	CH_3_	20.9	20.8	13.0	20.9	20.8	13.0
C-22	CH	78.3	78.3	75.0	78.3	78.3	75.0
C-23	CH_2_	27.3	27.3	25.2			25.2
C-24	CH_2_	42.3	42.2	42.2	42.3	42.2	42.2
C-25	C	71.3	71.1	71.1	71.3	71.1	71.1
C-26	CH_3_	28.9	29.0	29.0	28.9	29.0	29.0
C-27	CH_3_	29.7	29.3	29.3	29.7	29.3	29.3
C-1'	CH	103.3	102.1	102.1	103.3	102.1	102.1
C-2'	CH	74.6	74.9	74.9	74.6	74.9	74.9
C-3'	CH	77.5	77.6	77.6	77.5	77.6	77.6
C-4'	CH	71.2	71.29	71.3	71.2	71.29	71.3
C-5'	CH	78.1	78.1	78.1	78.1	78.1	78.1
C-6'	CH_2_	62.3	62.4	62.2	62.3	62.4	62.2
C-1"	C	131.0	130.4	130.4	132.1		131.2
C-2"	CH	115.9	112.3	112.3	117.8	114.9	114.8
C-3"	C	148.4	150.7	150.7	147.8		150.1
C-4"	C	148.7	149.5	149.5	146.8	147.9	148.9
C-5"	CH	117.9	117.1	117.1	117.6	117.0	117.0
C-6"	CH	122.2	123.4	123.4	122.7	124.8	124.7
C-7"	CH	146.0	145.8	145.8	143.5		143.8
C-8"	CH	117.3	117.5	117.5	119.9	119.4	119.5
C-9"	C	168.1	168.0	168.0	162.2		167.5
C-10"	CH_3_		56.7	56.7		56.3	56.7

* Signal not detected (too low concentration of the sample).

## 4. Conclusions

A chemical investigation of *Microsorum membranifolium* fronds was carried out within the framework of a thorough investigation of French Polynesia flora. This study showed that the fronds contained a new phytoecdysteroid, *E*-2-deoxy-20-hydroxyecdysone 3-[4-(1-β-D-glucopyranosyl)]-caffeate (**1**), together with the known compounds *E*-2-deoxy-20-hydroxyecdysone 3-[4-(1-β-D-glucopyranosyl)]-ferulate (**2**) and *E*-2-deoxyecdysone 3-[4-(1-β-D-glucopyranosyl)]-ferulate (**3**). In the same way, we identified their respective *cis* isomers **4**–**6** for the first time. Their structures were established on the basis of spectroscopic evidence. These results will lead us to find further novel secondary metabolites in Polynesian medicinal ferns.
